# Quercetin Alleviates Neuroinflammation in Chronic Insomnia by Modulating the RAGE/NF-κB Signaling Pathway: Insights from Network Pharmacology and In Vitro Validation

**DOI:** 10.3390/biomedicines14081766

**Published:** 2026-08-05

**Authors:** Guangming Liu, Nianshan Cai, Haiyi Wang, Miaomiao Liu, Wenjing Yan, Yiru Zhao, Meng Cui, Xiangpan Kong, Hongxu Sun, Peng Zhao

**Affiliations:** 1 Wuxi School of Medicine, Jiangnan University, 1800 Lihu Avenue, Wuxi 214122, China; lgm1628@126.com (G.L.); 6232811001@stu.jiangnan.edu.cn (N.C.); 6242811002@stu.jiangnan.edu.cn (H.W.); 6252811002@stu.jiangnan.edu.cn (M.L.); 6252811003@stu.jiangnan.edu.cn (W.Y.); 6232811004@stu.jiangnan.edu.cn (Y.Z.); x.kong@jiangnan.edu.cn (X.K.); 2 Pharmaceutical Basic Research Innovation Center for Gut Microbiota and Chronic Diseases, Ministry of Education, 1800 Lihu Avenue, Wuxi 214122, China; 3 Department of Psychiatry, Wuxi Mental Health Center, 156 Qianrong Road, Binhu District, Wuxi 214151, China; cmdoct@163.com; 4 University Hospital, Jiangnan University, 1800 Lihu Avenue, Wuxi 214122, China; sunhongxu336@jiangnan.edu.cn

**Keywords:** chronic insomnia, quercetin, neuroinflammation, RAGE/NF-κB axis, network pharmacology, BV2 microglia

## Abstract

**Background**: Chronic insomnia (CI) is increasingly recognized to be closely associated with neuroimmune dysregulation and neuroinflammation. While the dietary flavonoid quercetin exhibits known broad-spectrum anti-inflammatory properties, its specific multi-target network and underlying mechanisms concerning CI-associated neuroinflammation remain systematically unmapped. Therefore, this study integrated network pharmacology with in vitro experimental validation to elucidate the specific targets and mechanistic pathways of quercetin against neuroinflammatory responses implicated in CI. **Methods**: Potential targets of quercetin were predicted using the SwissTargetPrediction and SEA platforms, while CI-associated targets were curated from GeneCards, OMIM, and CTD. To bridge computational predictions with physiological relevance, protein–protein interaction (PPI) and functional enrichment analyses were integrated with molecular docking to assess the binding landscape of key candidates. Subsequently, to empirically validate these network-derived mechanistic hypotheses, in vitro experiments were conducted using an LPS-stimulated BV2 microglial model. Pro-inflammatory mediators were quantified via qRT-PCR, and the regulatory dynamics of the RAGE/NF-κB axis were evaluated by Western blotting. **Results**: Fifty-five overlapping targets were identified, prioritizing six hub genes (e.g., TNF, AKT1, IL6). By harmonizing the predicted network topology with experimental observations in the BV2 microglial framework, our results provide a unified mechanism linking quercetin to the suppression of central neuroinflammation. Enrichment highlighted the AGE-RAGE and IL-17 pathways as central mechanistic nodes. Molecular docking confirmed high-strength affinities between Quercetin and core targets. In the in vitro neuroinflammation model, quercetin (10, 30, and 60 μM) exerted a dose-responsive suppression of TNF-α, IL-1β, IL-6, and iNOS, while concurrently elevating anti-inflammatory IL-10 levels. Mechanistically, Quercetin significantly downregulated RAGE expression and blunted the phosphorylation of P65 and IκB, leading to significant reductions in p-P65/P65 and p-IκB/IκB ratios. **Conclusions**: Our findings demonstrate that Quercetin may attenuate neuroinflammatory responses associated with CI through modulation of the RAGE/NF-κB signaling axis, as indicated by network pharmacology prediction and further supported by validation in an LPS-stimulated BV2 microglial model. While these in vitro anti-inflammatory effects provide a robust mechanistic basis for targeting neuroimmune dysregulation, further in vivo behavioral studies are necessary to evaluate its direct therapeutic efficacy against chronic insomnia.

## 1. Introduction

Chronic insomnia (CI) is among the most common sleep disorders, characterized by persistent difficulty initiating or maintaining sleep with subsequent daytime impairment [1]. CI imposes a substantial individual and societal burden and is frequently associated with mood disorders, cardiometabolic dysregulation, and a reduced quality of life [2,3]. Current pharmacologic treatments are often limited by tolerance, dependence, residual sedation, and a high risk of relapse after discontinuation, underscoring the urgent need for safer and mechanism-informed adjunctive strategies [1,2,3]. Epidemiological evidence highlights that insomnia is highly prevalent, chronically persistent, and strongly linked to diverse psychiatric comorbidities and long-term adverse health outcomes [4,5].

Neuroinflammation refers to an inflammatory response within the central nervous system and is mainly mediated by resident glial cells, including microglia and astrocytes, together with cytokines, chemokines, reactive oxygen species, and other inflammatory mediators [6]. Accumulating evidence indicates that chronic neuroinflammation is a common pathological feature of neurodegenerative diseases, including Alzheimer’s disease and Parkinson’s disease, where sustained microglial activation and pro-inflammatory signaling contribute to neuronal injury and disease progression [7,8]. In the context of CI, sleep disturbance may promote inflammatory activation and alter cytokine rhythms, while elevated inflammatory mediators may further disturb sleep-regulatory networks, forming a bidirectional pathological loop between insomnia and neuroimmune dysregulation [9]. Chronic neuroinflammation is now recognized as a critical pathological axis linking central neurobehavioral deterioration with the progression of chronic neurodegenerative diseases. Emerging clinical and experimental evidence underscores that persistent glial overactivation creates a cytotoxic microenvironment, which not only accelerates neuronal loss in neurodegenerative conditions but also acts as a primary driver of sleep–wake regulatory failure. In the specific context of chronic insomnia (CI), this neuroinflammatory tone does not merely mirror sleep disturbance; rather, it functions as a central pathogenic mechanism. Continuous central nervous system inflammation fractures the neuroimmune rhythmicity of sleep-regulating nuclei, thereby sustaining sleep instability and impairing cognitive recovery [9,10]. Intestinal dysbiosis compromises gut barrier integrity, enabling the systemic translocation of microbial byproducts (such as lipopolysaccharides) that trigger peripheral immune responses, cross the blood–brain barrier, and alter the neuroimmune microenvironment of central sleep-regulatory centers. To uncover active agents within this axis, screening microbiota-associated metabolic profiles represents a logical strategy. Quercetin, a dietary polyphenol, is highly relevant here due to its unique reciprocal interaction with the gut ecosystem. Intestinal dysbiosis compromises barrier integrity, triggering systemic immune responses that alter the central neuroimmune microenvironment [11,12,13]. Within this axis, quercetin serves as a key exogenous substrate: it not only modulates gut microbial composition and repairs intestinal barrier integrity, but also undergoes extensive bacterial ring-cleavage biotransformation in the intestine. These derived small-molecule phenolic metabolites exhibit superior systemic signaling capacities, allowing them to traverse the blood–brain barrier and exert potent neuroprotective effects to intercept the inflammatory loops driving CI [14,15]. Given the inherent biological complexity and pleiotropic nature of the gut-microbiota-brain axis, mapping the complete mechanistic landscape requires an integrative systems-level approach [11,12,13]. Furthermore, while multiple signaling cascades participate in the pathogenesis and progression of CI, the AGE-RAGE axis and the NF-κB pathway represent critical immunological checkpoints [16,17]. The AGE-RAGE axis was prioritized in our study because it functions as a high-fidelity upstream “oxidative-inflammatory sensor” on microglial cells, providing a direct mechanistic link between systemic metabolic stress and central neuroinflammation. Although AGE-RAGE signaling is classically discussed in the context of diabetic complications [18]. and neurodegeneration [19]. Emerging neurobiological research has highlighted its role in sterile stress responses [20]. Under chronic sleep loss, central tissues experience intense oxidative stress, which accelerates the accumulation of danger signals (such as HMGB1 and S100B) in the brain [19]. RAGE functions as a versatile pattern recognition receptor on microglia that binds these non-AGE inflammatory ligands, triggering the phosphorylation of the downstream canonical NF-κB cascade [16,20]. This transcriptional activation drives a sustained surge of pro-inflammatory cytokines that directly injure sleep-regulating neural circuits, providing a key mechanistic rationale showing how metabolic stress translates into chronic neuroinflammation [19,20].

To decipher this intricate interplay among dietary phytochemicals, microbiota-driven biotransformation, and sleep regulation, we utilized a network pharmacology-based integrative framework [21]. This framework enabled the systematic identification of quercetin as a core active candidate, alongside dominant pathways—such as AGE-RAGE and NF-κB—involved in CI pathology. Following molecular docking simulations to assess structural binding configurations, we performed targeted in vitro experiments using BV2 murine microglial cells to harmonize in silico predictions with physiological observations. Based on this sequential design, we hypothesized that dietary-derived quercetin alleviates chronic insomnia-associated neuroinflammation by directly intercepting the microglial RAGE/NF-κB signaling cascade to suppress downstream cytokine transcription.

## 2. Materials and Methods

### 2.1. Databases and Computational Resources

The comprehensive list of data repositories, software versions, and computational platforms utilized in this study is systematically tabulated in Table 1.

### 2.2. Identification of Gut Microbial Metabolites and Potential Protein Targets

To ensure maximum clinical relevance and translational potential for human chronic insomnia, human genomic and proteomic databases were strictly utilized for the upstream in silico network pharmacology mapping. Murine BV2 microglial cells were subsequently chosen as a highly conserved mammalian in vitro system to validate the fundamental biochemical and regulatory dynamics of the predicted RAGE/NF-κB signaling axis.

The gut-MGene platform was utilized to harvest the comprehensive profiles of gut microbiota (GM) metabolites and their associated downstream targets. To identify these microbial metabolites, interaction data were filtered to include only records associated with “human” subjects. These metabolites were then mapped to their respective PubChem Compound Identifiers (CID) and Simplified Molecular Input Line Entry System (SMILES) codes. To optimize for computational modeling and structural integrity, we retained compounds with a SMILES string length of ≤200 characters.

For target forecasting, the canonical SMILES notations for the validated metabolites were input into the SEA (Similarity Ensemble Approach) and SwissTargetPrediction (STP) databases. For SEA results, only targets annotated as “HUMAN” were preserved. For STP results, a threshold of Probability > 0.1 was applied to ensure the accuracy of protein-metabolite interaction predictions [22]. Finally, the targets from both sources were merged, and duplicate entries were removed to construct the final metabolite-target interaction network. All data processing and filtering steps were conducted using R (version 4.3.3). Visualization was performed using the ggplot2 and ggvenn packages in R [23].

### 2.3. Screening of Pathological Targets Associated with CI

To identify CI-related genomic loci, data were aggregated from the GeneCards (Relevance score > 1), OMIM (approved symbol), and CTD (inference score 19.59) repositories. These candidate targets were then integrated within the RStudio environment, where intersectional analysis and graphical representation were executed utilizing the ggvenn and ggplot2 toolkits. The core targets modulating CI are constituted by the overlapping targets from GM, GutMgene, and CI [23].

### 2.4. Construction of the PPI Architecture

The intersectional targets were mapped onto the STRING database (medium confidence 0.400, hide disconnected nodes in the network) to generate the primary PPI framework. For subsequent network architecture visualization, Cytoscape (version 3.10.3) was employed. To recognize the key targets in the PPI network, we employed six algorithms: Degree, Stress, Radiality, MCC, MNC, and EPC. Targets satisfying these algorithmic criteria were prioritized for downstream investigation [24,25].

### 2.5. Functional Annotation and Pathway Enrichment Profiling

To derive mechanistic insights from the identified hub targets, functional enrichment analysis was performed utilizing the DAVID bioinformatics suite [26]. GO annotations spanned three fundamental domains: biological process (BP), cellular component (CC), and molecular function (MF). Simultaneously, KEGG pathway enrichment was implemented to delineate the underlying signaling architecture. Statistically significant terms were prioritized based on a combination of Gene count and −log10(*p* value), with a rigorous threshold of *p* < 0.05 applied for selection. The final enrichment landscapes were visualized through a specialized bioinformatics analysis platform-Wei Sheng Xin [27].

### 2.6. GeneMANIA-Based Target Expansion

Core targets were uploaded to GeneMANIA to obtain functionally associated genes (co-expression, physical interaction, pathway co-membership). Expanded targets were used for secondary enrichment and module analysis.

### 2.7. Module Identification (MCODE)

Expanded targets were re-submitted to STRING for constructing an expanded PPI network and then imported into Cytoscape. MCODE was applied to detect densely connected clusters (modules)using the following parameters: degree cutoff = 2, node score cutoff = 0.2, k-core = 2, and max depth = 5. These parameters were selected to match the network scale (111 nodes) and ensure module boundary specificity.

To prioritize biologically meaningful modules for downstream functional annotation, we applied a predefined inclusion criterion: MCODE score ≥ 4 and node count ≥ 4. Modules failing to meet this threshold were excluded from primary functional annotation; their complete statistics are provided in Supplementary Table S1. Modules satisfying these criteria were subjected to GO/KEGG enrichment analysis.

### 2.8. Computational Molecular Docking Simulations

The six consensus hub genes identified by intersecting all six CytoHubba algorithms in the PPI network are AKT1, TNF, IL6, IL1B, TP53, and PPARG. Among these, PPARG and AKT1 encode intracellular receptors/kinases that function as upstream master regulators of inflammatory signaling. Although TNF, IL-6, and IL-1β also ranked as core hubs, they are downstream effector cytokines whose transcriptional expression is governed by these upstream nodes. To ensure both topological centrality and biological relevance, candidate genes for molecular docking were selected using a dual-criterion approach: (i) inclusion in the six consensus hub genes, and (ii) high degree centrality in the exploratory computational network. Accordingly, molecular docking was focused on PPARG, AKT1, and IL-1β, with subsequent suppression of TNF, IL-6, and IL-1β validated by qRT-PCR.

To evaluate the binding complementarity between candidate proteins and metabolites, the three-dimensional structures of the former were acquired from the RCSB Protein Data Bank (PDB) (PPARG 8B95; IL-1β 6Y8I; AKT1 3CQW), whereas metabolite structures were sourced via the PubChem database (quercetin Compound CID: 5280343; daidzein Compound CID: 5281708; 3-(4-hydroxyphenyl) propionic acid Compound CID:10394; dihydrocaffeic acid Compound CID: 348154). Simulations were executed using the CDOCKER algorithm integrated within the Discovery Studio environment (BIOVIA). Receptor proteins and ligands were prepared using the Prepare Protein and Prepare Ligand protocols, respectively. The identification of binding coordinates was guided by co-crystallized ligand positions or validated active site residues. Scoring of docking configurations was based on CDOCKER interaction energy, with further qualitative assessment of binding motifs, including hydrogen bonding, π-stacking, and hydrophobic forces. A binding energy (BE) threshold of <0 kcal/mol served as the criterion for estimating the stability of the target-metabolite complex.

### 2.9. Drug-Likeness and ADMET Prediction

SwissADME and ADMETlab3.0 were used to assess drug-likeness, bioavailability, Lipinski compliance, and predicted toxicity signals (e.g., hepatotoxicity, h ERG risk, neurotoxicity, carcinogenicity).

### 2.10. Cell Culture and Treatments

Murine BV2 microglial cells (Procell, Wuhan, China, CL-0493) were cultured in DMEM high-glucose medium (Cytiva, Marlborough, MA, USA, SH30022), supplemented with 10% fetal bovine serum (FBS; BIOEXPLORER, Carolina, NC, USA, BS1615-110) and a 1% penicillin-streptomycin-amphotericin B solution (Biosharp, Beijing, China, BL142A). Propagation was conducted in a humidified incubator maintained at 37 °C with a constant 5% CO_2_ and 95% air atmosphere. The culture medium was refreshed every other day, and cells were subcultured upon reaching approximately 70% confluence, with passages 4 to 14 utilized for subsequent experiments.

For the experimental treatments, neuroinflammation was induced by exposing BV2 cells to lipopolysaccharide (LPS; Biosharp, Beijing, China, BS464-10 mg) at a final concentration of 100 ng/mL for 24 h. Quercetin (MERYER, Shanghai, China, M03006-10 G) was initially dissolved in sterile dimethyl sulfoxide (DMSO) to prepare a concentrated master stock solution, which was subsequently diluted in fresh culture medium to achieve the desired working concentrations; the final concentration of the DMSO vehicle was strictly restricted to <0.1% (*v*/*v*) across all treatment groups to eliminate potential vehicle-induced cytotoxicity. To establish non-toxic therapeutic dosing thresholds, cell viability was evaluated using a CCK-8 colorimetric assay kit (Vazyme, Nanjing, China, A311-01). Cells were seeded in 96-well plates and exposed to varying concentrations of Quercetin (10, 30, 60, and 100 μM) for 24 h, after which the optical density at 450 nm was recorded to calculate survival percentages [28]. All cellular assays and subsequent molecular measurements were performed with a minimum of three independent biological replicates (*n* = 3).

Furthermore, transcriptional profiling of pro-inflammatory markers (e.g., TNF-α, IL-1β, IL-6, and iNOS) was performed via qRT-PCR (Table 2). Target gene expression was normalized to the endogenous control GAPDH using the comparative 2^−ΔΔCt^ method [29]. For proteomic analysis, fractions were isolated and quantified utilizing a colorimetric BCA assay (Beyotime, Shanghai, China, P0012S). Equivalent samples underwent electrophoretic separation via SDS-PAGE (Beyotime, P0015) and were immobilized onto PVDF membranes (Merck, Darmstadt, Germany, IPVH00010). Following passivation with a BSA solution, primary antibody probing (RAGE, p-P65, total P65, p-IκB, and total IκB) was conducted at 4 °C for an overnight period. Immunoreactive bands were visualized through ECL (Theorell, Wuhan, China, TALR-001), and optical densities were assessed using ImageJ(v2024). Activation of the RAGE/NF-κB axis was quantified by calculating phosphorylation-to-total protein quotients, normalized to β-actin (SAB, 41456, 1:10000)[19,30]. The primary antibodies and their dilution ratios were as follows: RAGE (Abcam, Cambridge, UK, ab216329, 1:1000), p-P65 (Abcam, ab76302, 1:1000), total P65 (Abcam, ab32536, 1:1000), p-IκB (Abcam, ab92700, 1:1000), total IκB (Abcam, ab32518, 1:1000), and β-actin (SAB, 41456, 1:10,000).

### 2.11. Statistical Analysis

All data are presented as mean ± standard error. Statistical analysis was performed using GraphPad Prism software (v10.1.2). Normality of the data distribution was first assessed using the Shapiro–Wilk test. For comparisons among three or more groups, one-way analysis of variance (one-way ANOVA) was adopted if the data passed the normality test, followed by Tukey’s post-hoc test for further pairwise comparisons. Non-parametric analysis using the Kruskal–Wallis’s test was applied to data that violated the assumption of normality. The statistical significance level was set at *p* < 0.05, and a *p* value less than 0.05 was considered statistically significant.

## 3. Results

### 3.1. Identification of Consensus Targets for GM-Metabolites and CI

**Figure 1:**
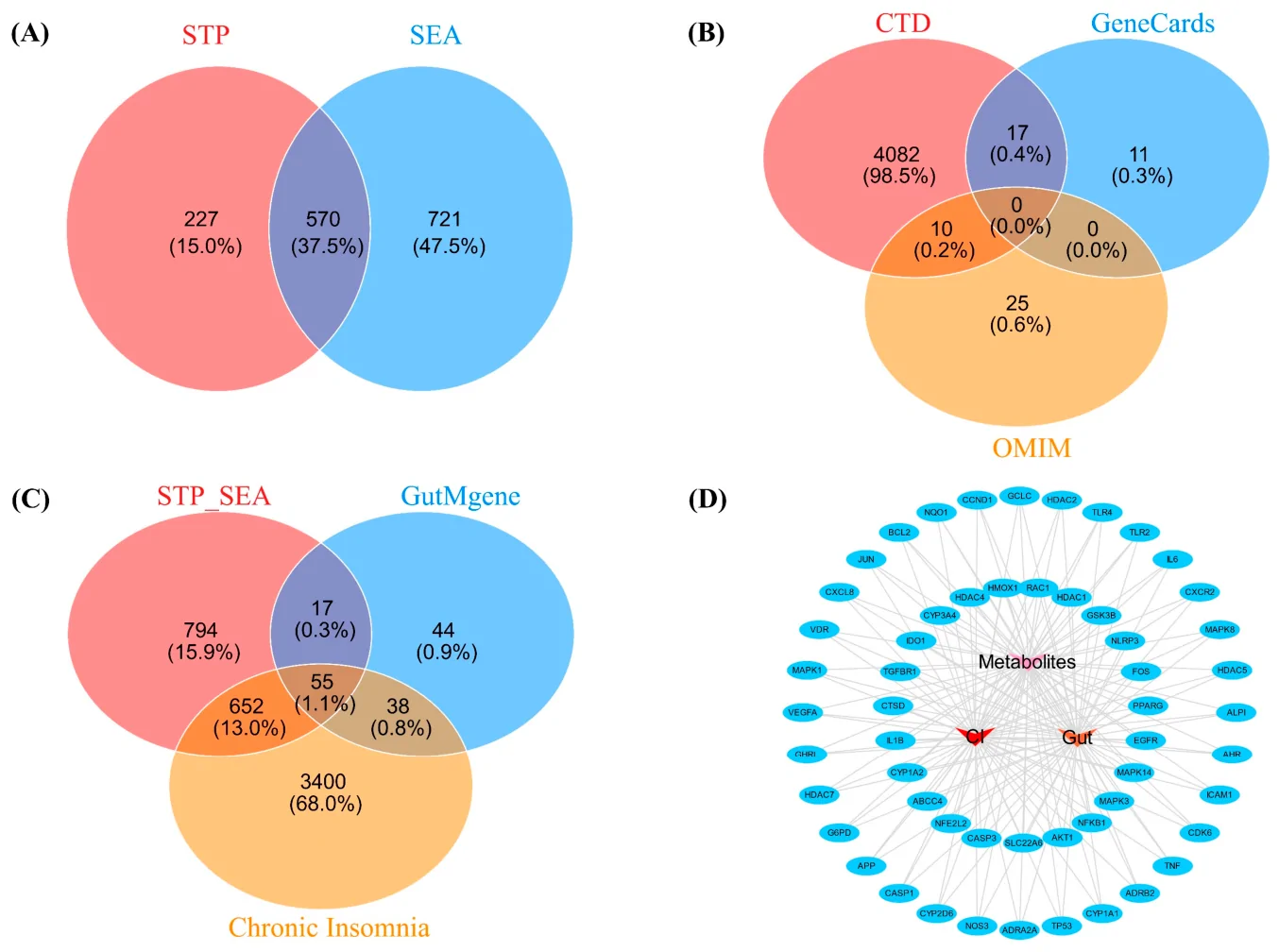
Systematic acquisition of therapeutic targets. (**A**) Cross-platform targets for gut microbial metabolites identified via SEA and STP. (**B**) Profile of CI-associated targets curated from GeneCards, OMIM, and CTD. (**C**) Identification of overlapping core targets shared among gut microbiota, metabolites, and CI pathology. (**D**) Integrated network architecture of the Gut-Metabolite-CI-Target axis.

Initial extraction from the SEA and STP databases yielded 1518 microbiota-associated targets (Figure 1A). After merging and deduplicating, 4145 CI-related targets were obtained from GeneCards, OMIM, and CTD (Figure 1B). The intersection of the gut metabolite targets, CI targets, and gut microbiota targets produced 55 common targets (Table S2), which were designated as key mediators in CI progression (Figure 1C). To clarify their complex relationships, we then assembled a gut–metabolite–target–disease network (Figure 1D).

### 3.2. Topological Prioritization of Hub Nodes Within the PPI Interactome

**Figure 2:**
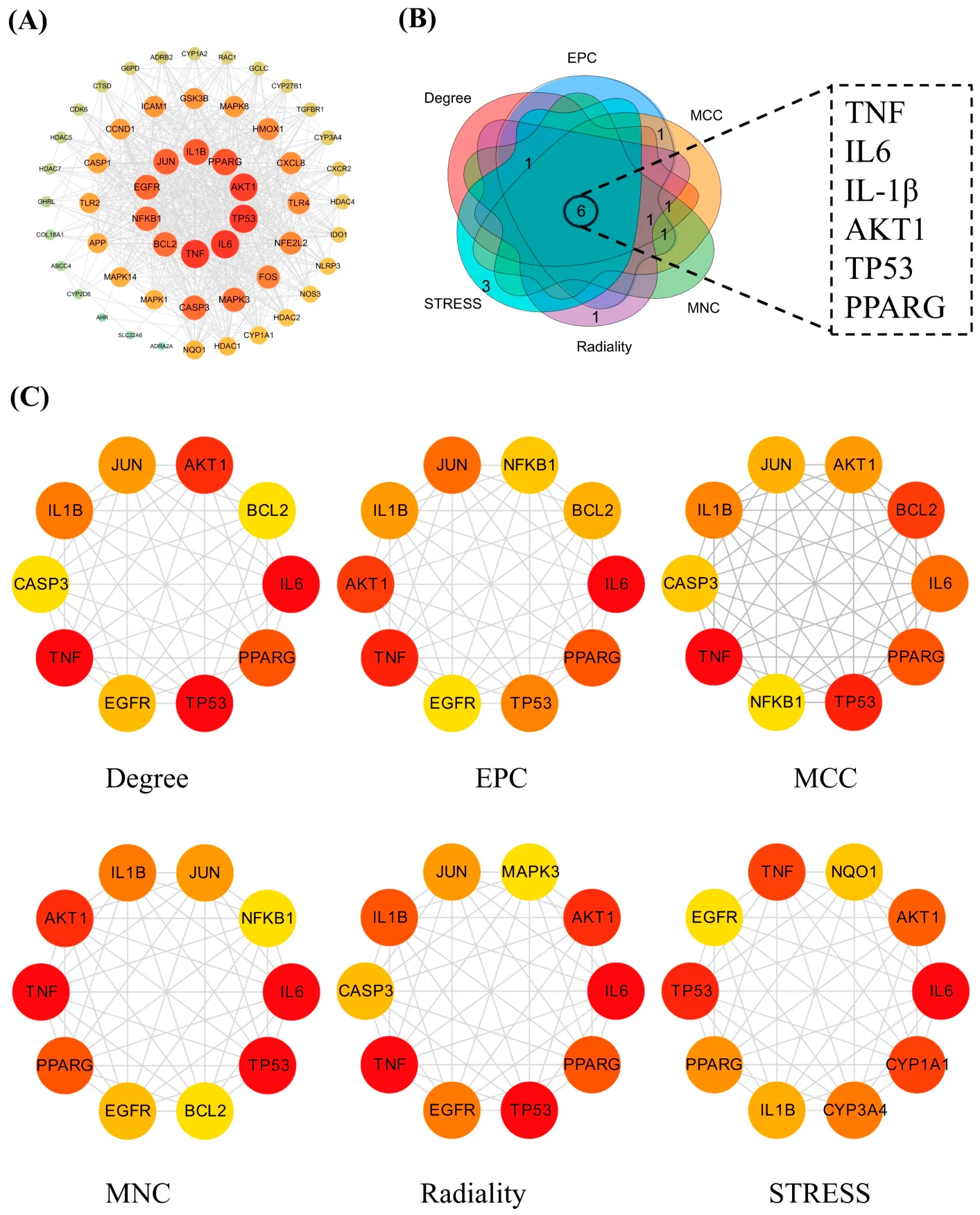
The identification of core targets. (**A**) Protein–protein interaction (PPI) network of overlapping targets. Nodes represent proteins; edges represent protein–protein associations. Node colors indicate degree values (from green to red, with darker colors representing higher degrees). (**B**) Venn diagram showing the core targets commonly identified by six algorithms. TNF: Tumor Necrosis Factor, IL6: Interleukin 6, IL-1β: Interleukin 1 Beta, AKT1: AKT Serine/Threonine Kinase 1, TP53: Tumor Protein P53, PPARG: Peroxisome Proliferator-Activated Receptor Gamma. (**C**) Identification of core targets using algorithms from the CytoHubba plugin in Cytoscape. Nodes are colored according to MCC scores, with colors ranging from red (highest score) to yellow (lower scores).

To delineate pivotal therapeutic targets for chronic insomnia (CI), 55 interventional targets were integrated into the STRING database to construct a sophisticated protein–protein interaction (PPI) network. The resulting interactome, characterized by 54 nodes and 637 edges, was visually rendered and interrogated using Cytoscape 3.10.3 (Figure 2A). To objectively distill essential hub genes, the network topology was rigorously evaluated across six CytoHubba algorithmic dimensions: Stress, Degree, Radiality, MCC, MNC, and EPC. An intersectional analysis of the top 10 candidates ranked by each method (Figure 2C). six consensus hub targets were identified: AKT1, TNF, IL-6, IL-1β, TP53, and PPARG (Figure 2B). These nodes represent the core regulatory junctions within the CI-related neuroimmune network.

### 3.3. Functional Characterization via GO and KEGG Enrichment

**Figure 3:**
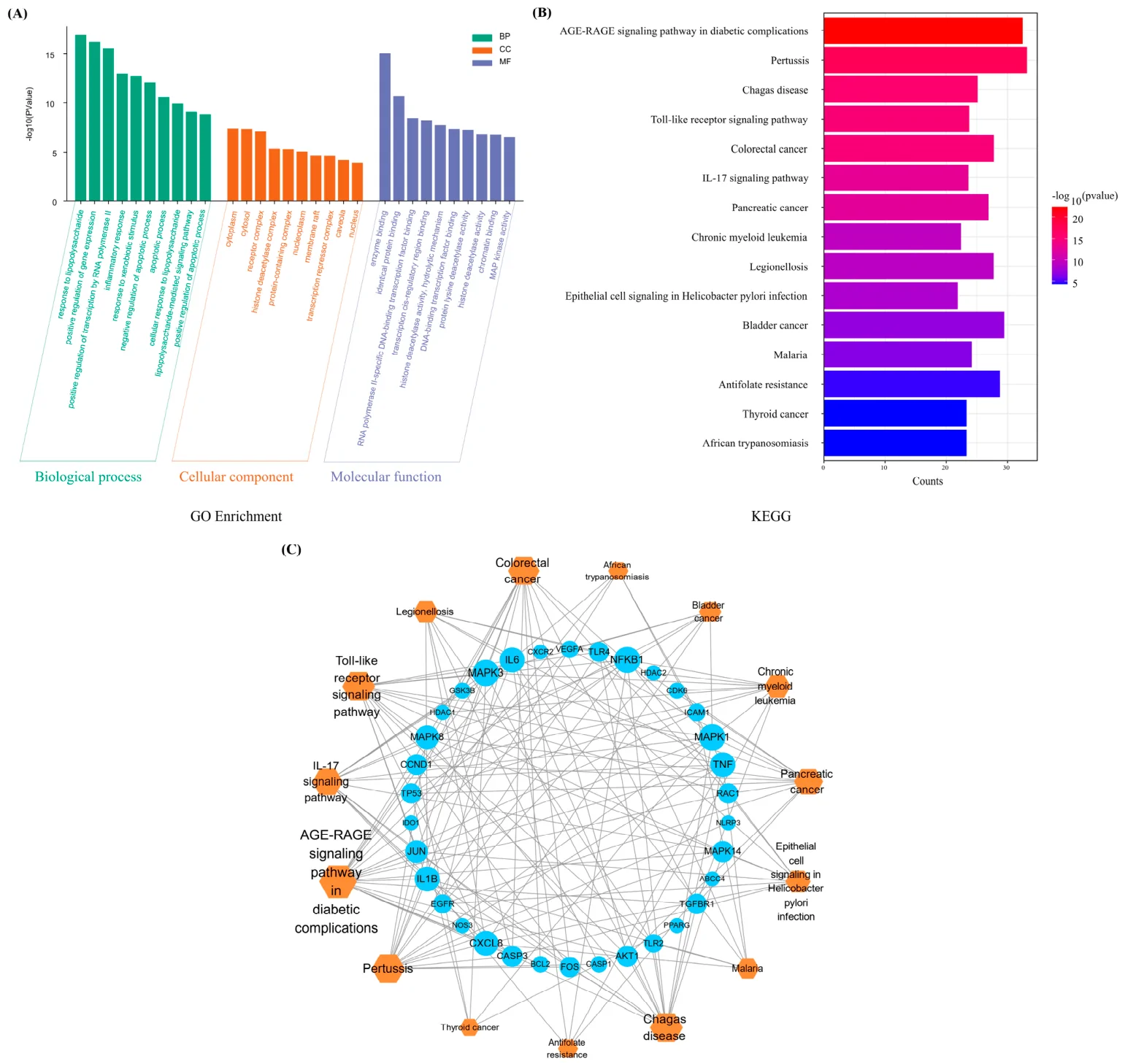
Functional landscape and biological enrichment of gut microbiota-derived metabolites in CI. (**A**) Distribution of the top 10 enriched terms across biological process (BP), cellular component (CC), and molecular function (MF) domains, ranked by statistical significance. (**B**) Identification of the top 15 KEGG pathways prioritized by *p*-value significance. (**C**) Topological interconnectivity and regulatory architecture of the target-pathway network.

To elucidate the biological landscape through which gut microbiota-derived metabolites influence CI, the 55 interventional targets were analyzed using the DAVID bioinformatics resource. For Gene Ontology (GO) annotation, terms significantly enriched within the BP, CC, and MF categories were screened using a stringent threshold of FDR-adjusted *p* < 0.05 (Figure 3A). Our analysis revealed that these core targets are predominantly involved in inflammatory responses, apoptotic modulation, and cellular reactions to lipopolysaccharides. Furthermore, KEGG enrichment (Figure 3B) unveiled the pivotal involvement of these targets in the IL-17 and AGE-RAGE signaling cascades, alongside the Toll-like receptor pathway. An integrated target-pathway interactome (Figure 3C) underscores the therapeutic potential of these metabolites to mitigate CI by recalibrating immune-related signaling and inflammatory homeostasis.

### 3.4. Construction of the Functional Association and GMFA Network for Prioritized Hub Targets

**Figure 4:**
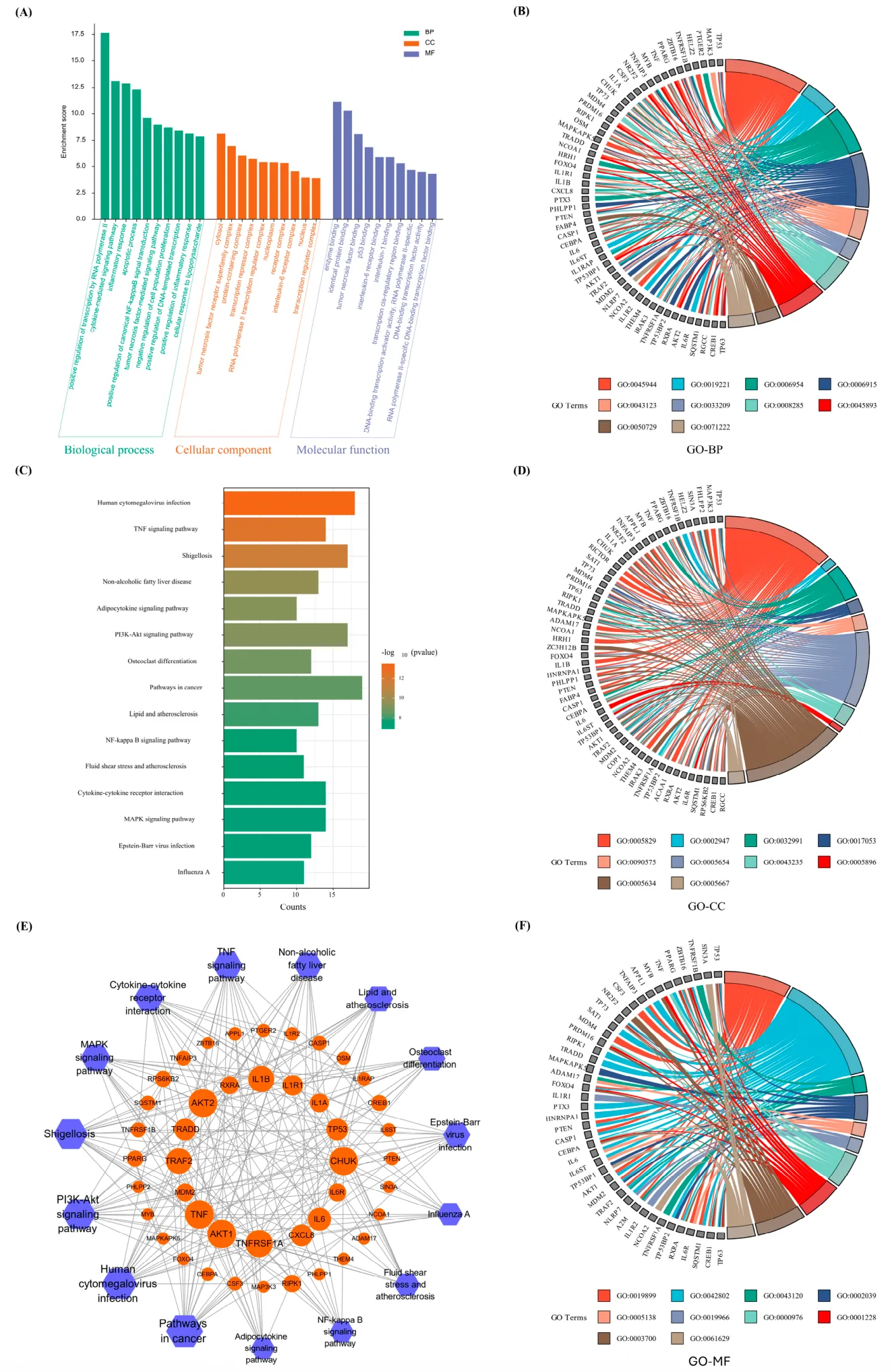
Functional association network analysis (GMFAN) of the six primary hub targets (**A**–**F**) via GeneMANIA.

To delineate functional interconnections and identify peripheral genes associated with the six hub genes, the GeneMANIA database was utilized. For each primary gene, an additional ten neighbors were incorporated based on their physical interactions, co-expression patterns, and shared genetic associations (Figure 4A–F). This strategy yielded a broadened gene interaction network, facilitating the identification of candidates with superior therapeutic relevance.

### 3.5. Functional Enrichment Profiling via GO and KEGG for GMFA Targets

**Figure 5:**
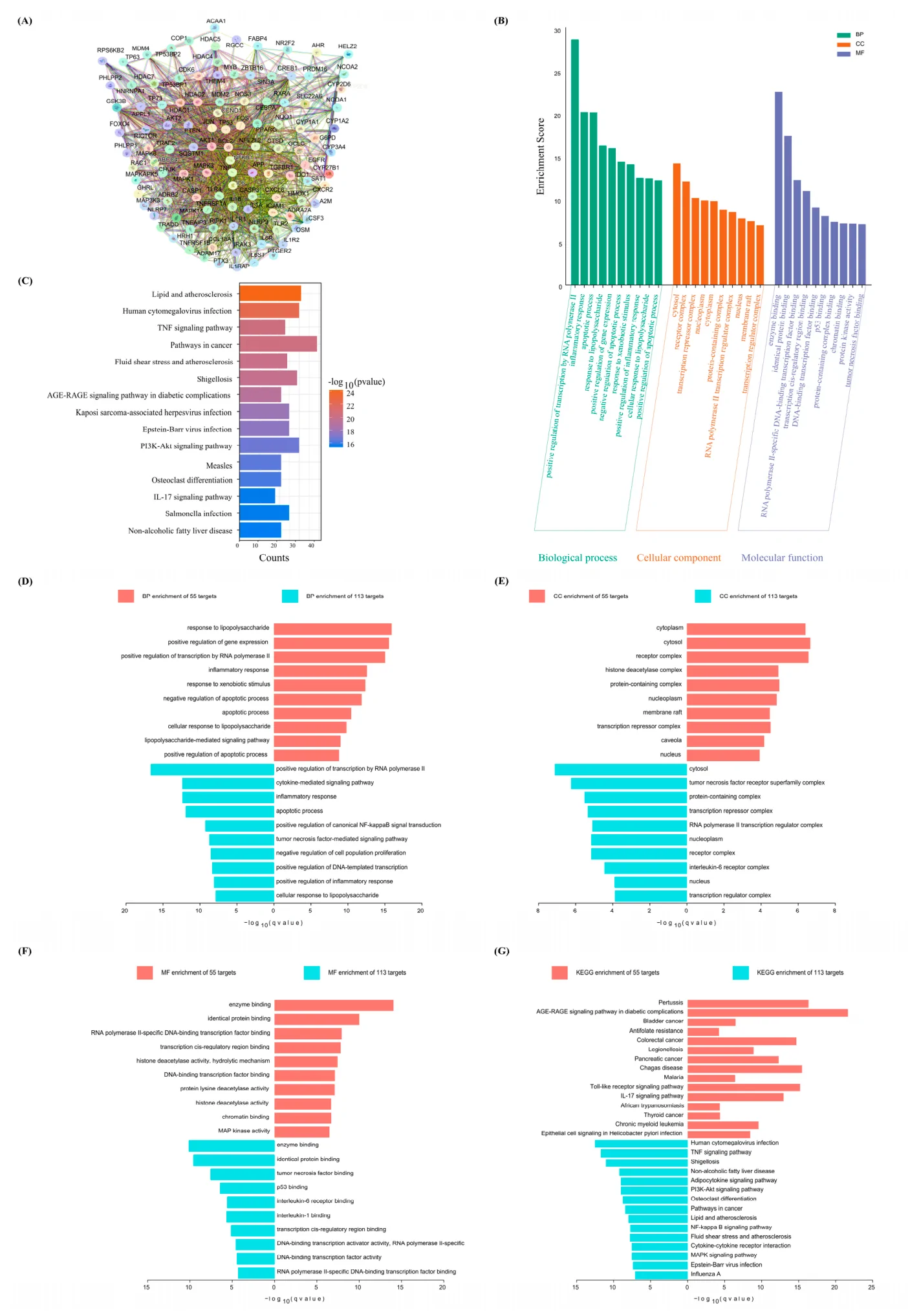
Comprehensive GO and KEGG profiling for the expanded target set. (**A**) Visualization of the PPI interaction network for the expanded core targets. (**B**) Selection of the 10 most enriched BP, CC, and MF categories sorted by *p*-value significance. (**C**) Leading 15 KEGG pathways revealed by pathway mapping. (**D**–**F**) Cross-set evaluation comparing GO domains (BP, CC, and MF) across the 55-target and expanded-target datasets. (**G**) Enrichment pattern comparison for KEGG pathways, highlighting the functional consistency between the hub targets and the expanded cohort.

**Figure 6:**
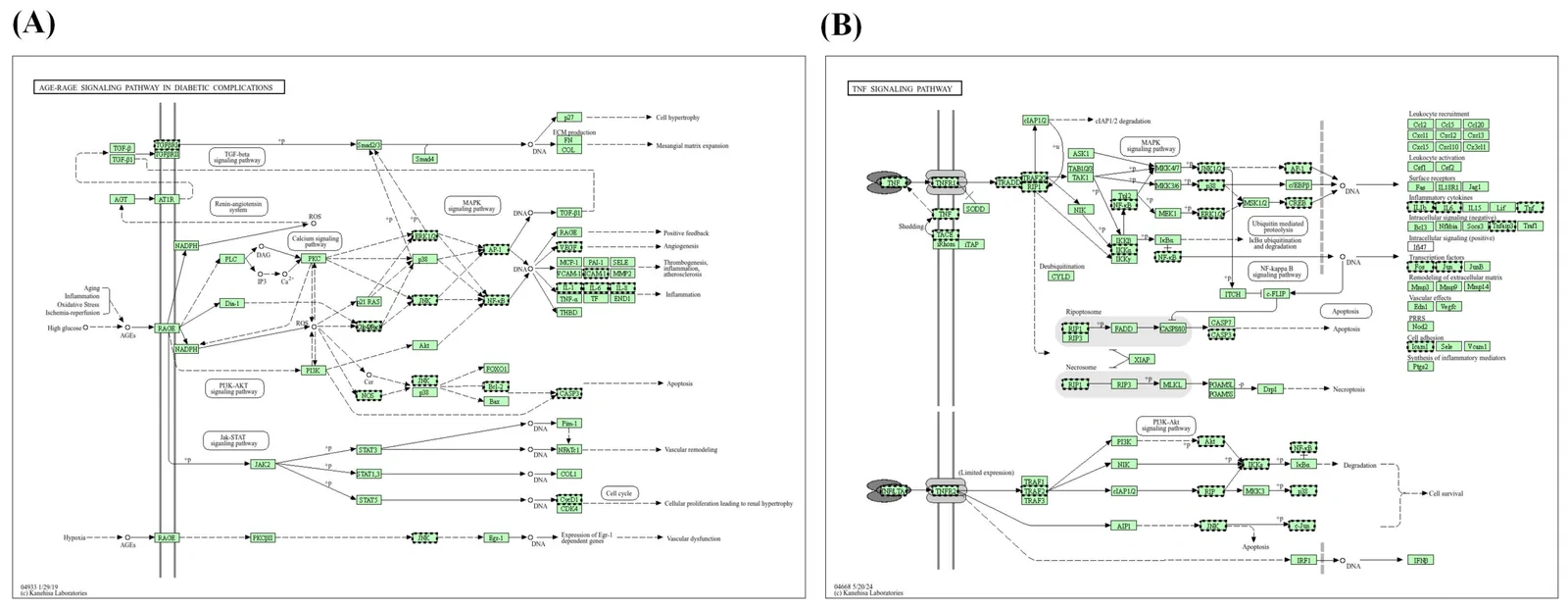
Key molecular signaling pathways curated for CI. (**A**) The AGE-RAGE signaling pathway involved in diabetic sequelae. (**B**) Canonical TNF signaling pathway architecture illustrating target interactions. Arrows and symbols are defined as follows: solid lines with arrowheads (→) denote direct protein–protein interactions or enzymatic phosphorylation; dashed lines (--→) denote indirect regulation or nuclear translocation of transcription factors; blunted lines (⊥) denote inhibitory effects; the symbols +P and -P indicate the phosphorylated and unphosphorylated states of the protein, respectively.

To delineate the biological implications of the GMFA dataset, we consolidated 55 primary nodes into an expanded interactome of 113 unique targets (Figure 5A). Subsequent GO and KEGG mapping was executed to delineate the functional architecture of this broadened network. Biological process (BP) annotations underscored a concentrated regulatory influence on cytokine-mediated signaling and inflammatory modulation (Figure 5B). Concurrently, cellular component (CC) and molecular function (MF) profiles highlighted prominent engagement with protein-receptor complexes and cytokine sequestration, involving mediators such as TNF, IL-6, and IL-1β. To assess the phenotypic stability of these functional clusters, a comparative mirror-plot analysis was performed between the core (55 targets) and expanded (113 targets) datasets (Figure 5D–G). This side-by-side visualization revealed a remarkable concordance in enrichment hierarchies; for instance, inflammatory cascades and apoptotic signaling consistently ranked as top-tier BP terms in both cohorts (Figure 5D). Furthermore, while the expanded 113-target set yielded finer granularity for pathways like NF-κB and PI3k/Akt signaling (Figure 5C), the core AGE-RAGE and TNF axes remained conserved central nodes (Figure 6A,B). Such cross-scale validation reinforces the reliability of our bioinformatic model, illustrating the stable regulatory bridge through which gut microbiota-derived metabolites modulate CI pathophysiology.

### 3.6. Functional Clustering Analysis

**Figure 7:**
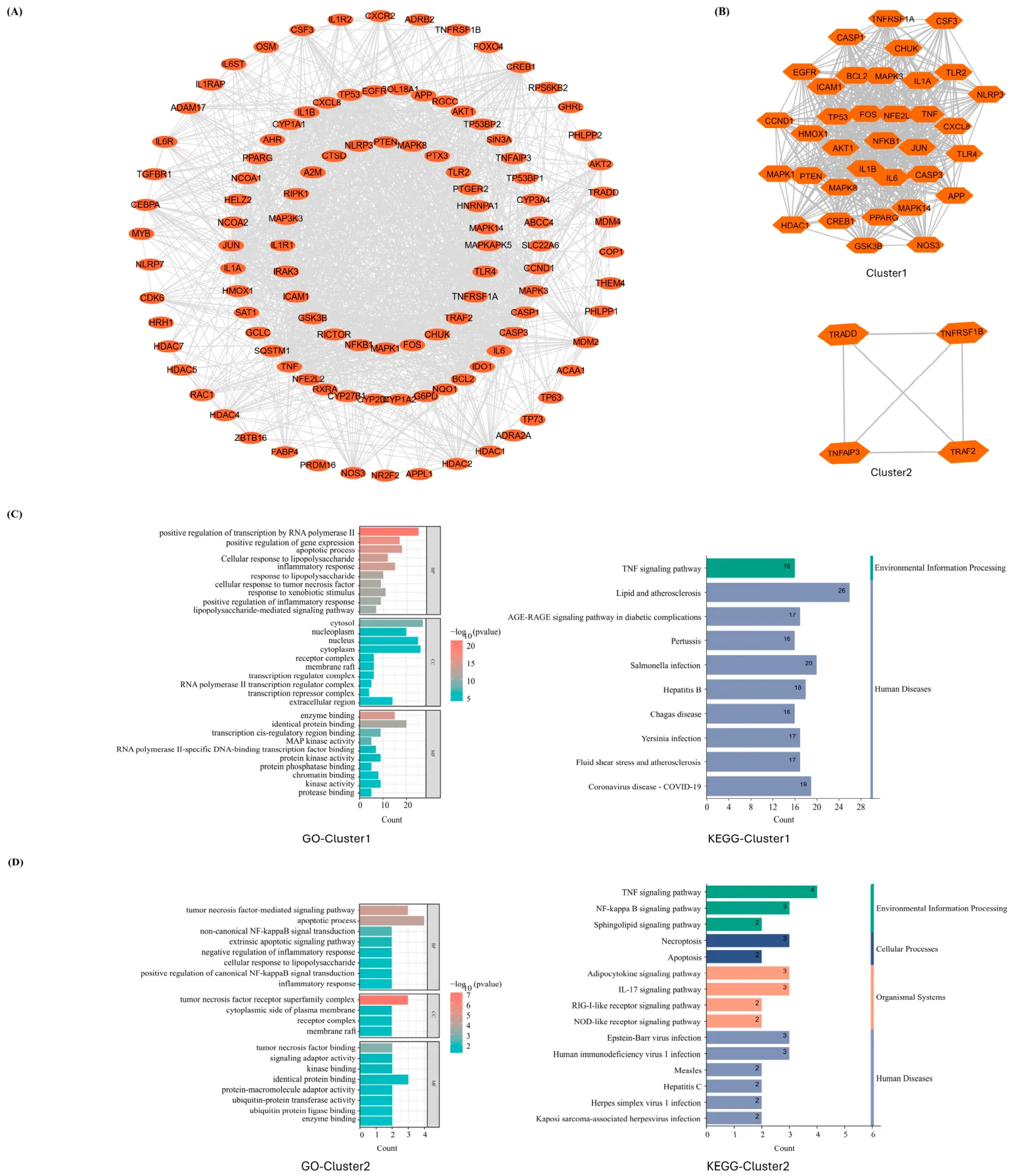
Modular network partitioning and functional characterization within the GMFAN. (**A**) Visualization of the protein–protein interaction (PPI) architecture for the expanded core target set. (**B**) Identification of the two most prominent functional modules via MCODE clustering. (**C**) Systematic GO and KEGG functional annotation for Cluster 1. (**D**) Enrichment profiling of biological processes and pathways associated with Cluster 2.

To further dissect the mechanistic roles of gut microbiota-derived metabolites in CI, we performed modularity analysis by integrating the 113 identified targets into the STRING database, followed by topological clustering via the MCODE algorithm. After pruning isolated nodes, the resulting GMFA PPI network comprised a robust architecture of 111 nodes and 1467 interactions (Figure 7A). MCODE-based partitioning yielded two distinct functional modules: Cluster 1, a high-density cluster (35 nodes, 528 edges; score = 31.059), and Cluster 2, a smaller but significant unit (4 nodes, 6 edges; score = 4.0) (Figure 7B). Subsequent GO and KEGG enrichment analyses prioritized common functional themes across both clusters, including apoptotic pathways, inflammatory responses to lipopolysaccharides, and diverse protein-binding activities (Figure 7C). Notably, KEGG profiling categorized these clusters into functional hierarchies—ranging from environmental information processing to organismal systems and disease-related pathways (Figure 7C,D). These modular findings highlight the multi-layered regulatory influence of metabolites on CI.

Furthermore, the identified pathways encompass critical signaling axes—notably the TNF, IL-17, and NF-κB cascades—which function as pivotal drivers in both the initiation and pathological evolution of the disease. Taken together, these data provide a definitive mechanistic landscape through which microbial metabolites exert their bioactivity, elucidating the interconnected biological processes and identifying potential nodes for therapeutic intervention. By mapping these specific molecular interactions, this study significantly refines our grasp of the underlying pathophysiology and establishes a robust framework for subsequent clinical and experimental validation.

### 3.7. A Hypothesis-Generating Computational Framework of Microbiota-Substrate-Metabolite-Target Associations

**Figure 8:**
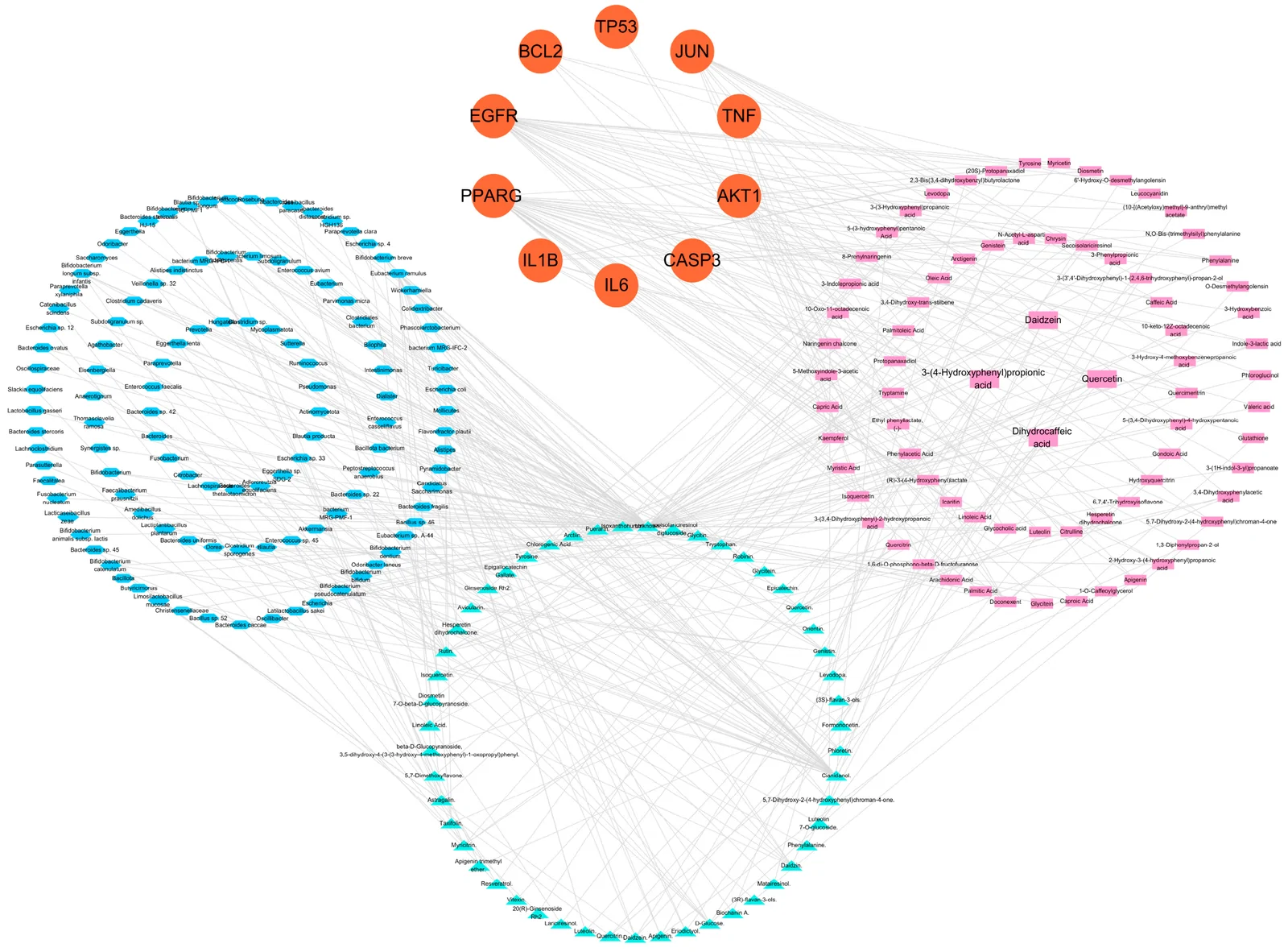
A hypothesis-generating computational network for future microbiome–metabolome validation.

To elucidate the therapeutic bridge between core gut microbiota and CI, an integrative “microbiota–substrate–metabolite–target” interactome was constructed to map their sophisticated interdependencies. As illustrated in Figure 8, the network architecture is visually color-coded: gut taxa (blue), metabolic substrates (cyan), derived metabolites (pink), and target genes (orange). This comprehensive landscape, encompassing 10 targets, 117 microbial taxa, 51 substrates, and 76 metabolites across 410 edges, underscores a highly connected regulatory system. Specifically, PPARG, EGFR, and AKT1 emerged as the most densely connected protein hubs. Meanwhile, compounds such as quercetin, 3-(4-hydroxyphenyl) propionic acid, daidzein, and dihydrocaffeic acid exhibited the widest taxonomic associations, pointing to their potential as pivotal functional modulators in CI. Topological analysis via Cytoscape identified these specific metabolites and targets as central nodes, with the aforementioned compounds and genes achieving the highest degree centrality scores within their respective categories.

**Figure 9:**
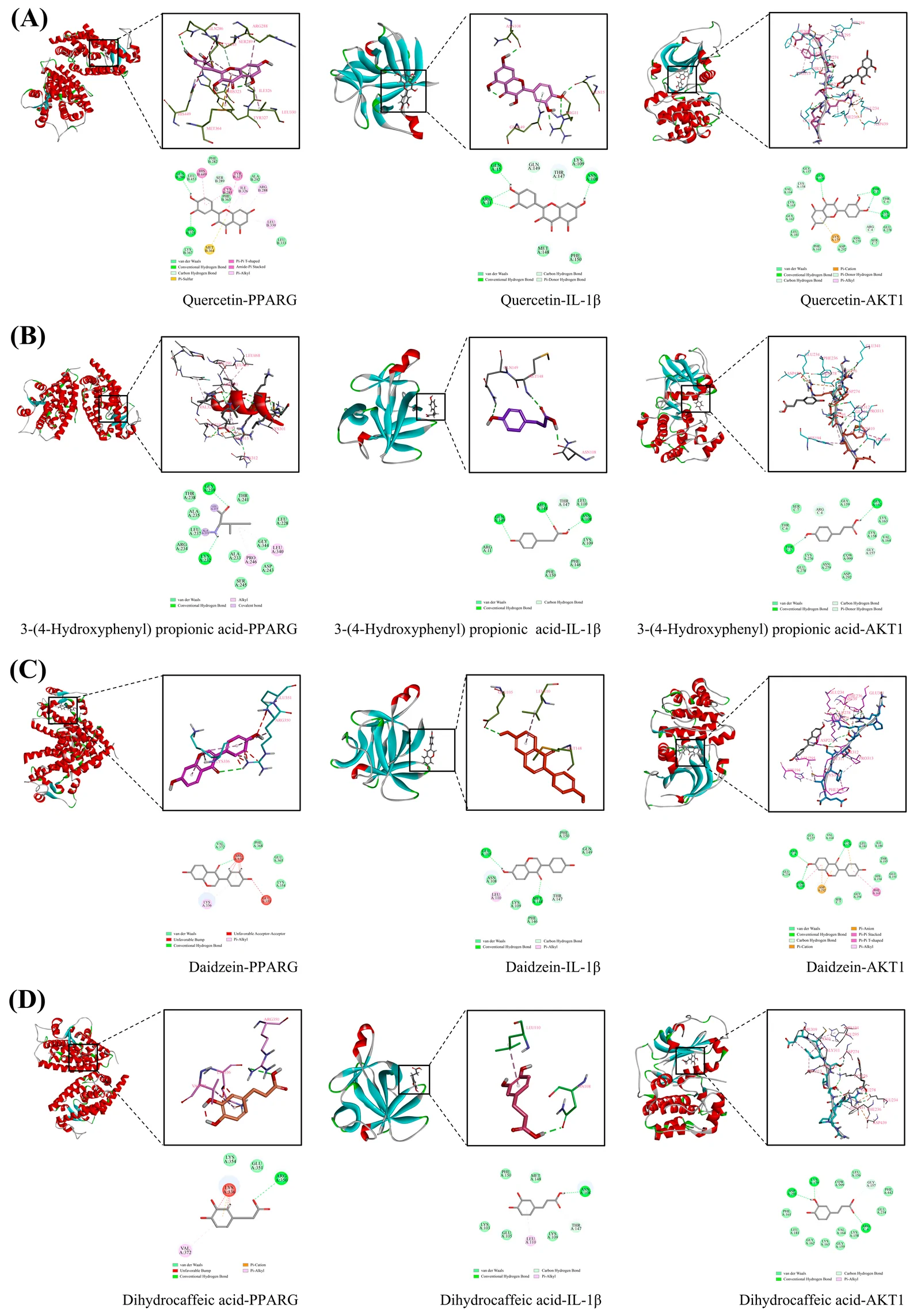
Structural visualization of molecular docking interactions. (**A**–**D**) Representative docking configurations of four candidate metabolites—Quercetin, 3-(4-hydroxyphenyl) propionic acid, Daidzein, and Dihydrocaffeic acid—with core protein targets (PPARG, IL-1β, and AKT1), respectively. The binding poses illustrate the precise spatial orientation within the target active sites.

The binding landscapes between the identified hub targets and key metabolites were rigorously interrogated via molecular docking simulations. Thermodynamic assessments revealed that all protein-ligand pairs achieved binding energies of less than 0 kcal/mol, indicating spontaneous molecular recognition; notably, values falling below the −5 kcal/mol threshold signified particularly robust interactions (Table 3 and Table S3). Quercetin emerged as the candidate with the highest affinity, consistently surpassing the 5 kcal/mol benchmark. Representative structural orientations and docking poses are illustrated in Figure 9A–D. Furthermore, ADMET profiling (Table 4 and Table 5) established that all four metabolites possess drug-like characteristics, including optimal bioavailability and strict adherence to Lipinski’s Rule of Five. Safety assessments confirmed no significant risk of cardio- or hepatotoxicity, though a mild carcinogenic potential was associated with quercetin and daidzein. In this regard, it is important to acknowledge that the mild carcinogenic potential of daidzein may be associated with its known estrogenic activity, a factor that necessitates careful consideration in clinical applications. In contrast, 3-(4-hydroxyphenyl) propionic acid and dihydrocaffeic acid exhibited no such risks. Taken together, these data underscore the potent interactions between key metabolites and core targets, validating their pharmacological relevance in CI.

### 3.8. Quercetin Attenuates LPS-Triggered Neuroinflammation in BV2 Microglia via RAGE/NF-κB Inhibition

**Figure 10:**
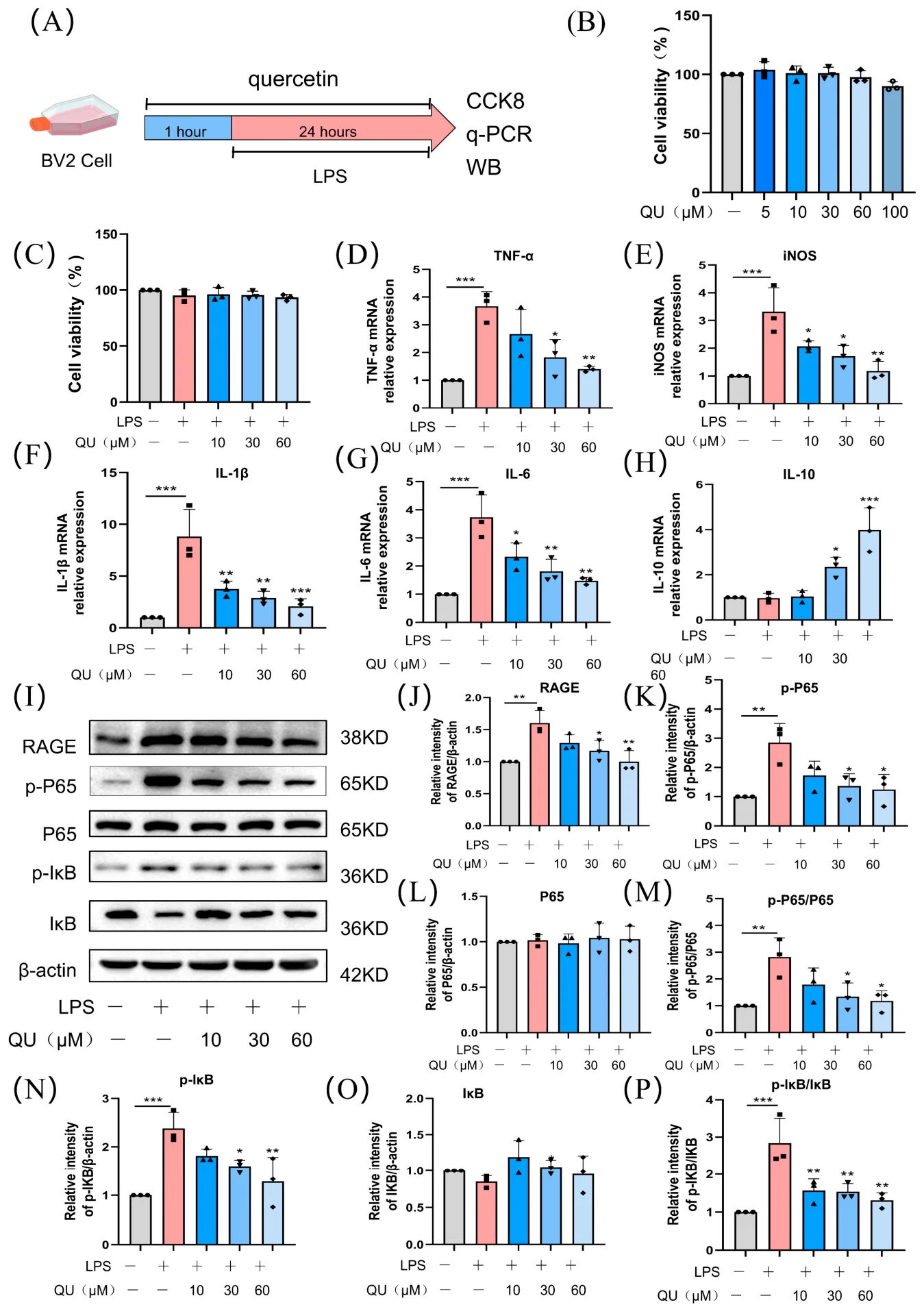
Regulatory impact of QU (quercetin) on inflammatory mediators and RAGE/NF-κB signaling in LPS-primed BV2 cells. (**A**) Schematic outline of the experimental timeline. (**B**) Assessment of cell viability via CCK-8 assay after a 24-h incubation with Quercetin (0–100 μM, *n* = 3). (**C**) Impact of pre-treating BV2 cells with Quercetin (0–60 μM) LPS stimulation (100 ng/mL) on overall survival. (**D**–**H**) Quantitative PCR profiling illustrating the mRNA expression of pro-inflammatory cytokines (TNF-α, iNOS, IL-1β, IL-6) and the anti-inflammatory mediator IL-10, normalized against the control. (**I**) Representative immunoblot images displaying RAGE, total, and phosphorylated forms of P65 and IκB across different treatment groups. (**J**–**P**) Densitometric quantification of protein expression relative to β-actin (*n* = 3). Values are presented as mean ± SEM of three autonomous biological replicates. * *p* < 0.05, ** *p* < 0.01 and *** *p* < 0.001 determined by one-way ANOVA with Tukey’s post hoc test.

To ensure that the observed anti-inflammatory effects were not biased by cytotoxicity, a CCK-8 assay was initially conducted. The results confirmed that neither Quercetin (QU, 5–100 μM) nor 100 ng/mL LPS compromised BV2 cell viability over a 24-h period (Figure 10B, C). Following the experimental protocol (Figure 10A), cells were primed with QU for 1 h before LPS stimulation. Transcriptional analysis via qRT-PCR revealed that LPS triggered a robust upregulation of pro-inflammatory cytokines, including TNF-α, iNOS, IL-1β, and IL-6 (*p* < 0.001, Figure 10D–G). Conversely, QU pre-treatment dose-dependently suppressed these markers while concurrently elevating the anti-inflammatory cytokine IL-10 (Figure 10H). Western blotting (Figure 10I) demonstrated that QU effectively inhibited LPS-induced RAGE expression and suppressed the phosphorylation of P65 and IκB (Figure 10J,K,N). Significant reductions in pP65/P65 and pIκB/IκB ratios were quantified (Figure 10M,P), without altering total protein levels (Figure 10L,O). Collectively, these findings suggest that Quercetin alleviates microglial neuroinflammation by modulating the RAGE/NF-κB signaling axis.

## 4. Discussion

In the current study, an integrative framework encompassing network pharmacology, topological module analysis, and in silico molecular docking was implemented to decode the mechanistic nexus among dietary phytochemicals, gut microbiota-driven biotransformation, and chronic insomnia (CI) [23,31]. Our multidimensional findings consistently converge on a central theme: the interplay between immune-inflammatory activation and immuno-metabolic homeostasis. The reciprocal relationship between sleep hygiene and immune function remains a cornerstone of neurobiology, where pro-inflammatory cytokines function as pivotal sleep-regulatory mediators capable of modulating sleep–wake stability [5,9]. By prioritizing core hub targets. we demonstrated that the systemic context of CI is likely orchestrated by cytokine-driven inflammation and metabolic checkpoints, rather than suggesting a direct cure for the sleep disorder itself [3,32]. We hypothesize that perturbations in gut barrier integrity enhance the systemic translocation of microbial byproducts (e.g., LPS), which in turn amplifies innate immune sensing and reshapes the neuroimmune environment essential for healthy sleep regulation [11,12,13,33,34].

The emergence of TNF, IL6, and IL1B as network hubs reflects a feed-forward inflammatory structure that may contribute to CI persistence [35,36,37,38]. These pathways channel into NF-κB-dependent transcriptional landscapes, fostering a state of sustained, low-grade systemic inflammation that links sleep disturbance with broader immuno-metabolic dysfunction [16,39]. A significant discovery in this network was the prominence of AGE–RAGE signaling [17,18]. Although this pathway is most classically discussed in the context of diabetic and metabolic disorders, its relevance to chronic insomnia is increasingly supported by neurobiological research [20]. Emerging evidence suggests that sleep deprivation induces systemic and central oxidative stress, which accelerates the formation and accumulation of advanced glycation end products (AGEs) in the brain [19]. The binding of these AGEs to RAGE on microglial cells triggers robust NF-κB-dependent neuroinflammation. Therefore, highlighting the AGE-RAGE pathway in this study provides a crucial mechanistic rationale for how sleep loss-induced metabolic stress translates into chronic neuroinflammation, further disrupting sleep architecture. By potentiating oxidative-inflammatory tone, AGE–RAGE sensitizes inflammatory circuits, thereby reinforcing a biological environment unfavorable for sleep restoration [20].

To counter this immuno-metabolic dysfunction, our network topology prioritized quercetin, a well-known dietary flavonoid, alongside microbially derived metabolites such as 3-(4-hydroxyphenyl) propionic acid and daidzein [40,41]. These candidates align with a diet–microbiota paradigm. Rather than being a direct microbial metabolite, quercetin is a plant-derived polyphenol that extensively interacts with the gut microbiome; it shapes microbial composition and undergoes microbial biotransformation into bioactive derivatives capable of systemic signaling [42]. Docking analysis provided additional evidence for the structural plausibility of compound-target interplay, showing favorable binding energies against prioritized targets like AKT1 [43]. However, as is a universally accepted limitation of such in silico methodologies, molecular docking exclusively predicts structural binding potential [24]. It cannot definitively confirm biological activity, functional target engagement, or in vivo therapeutic effects [44]. Nonetheless, the integration of network centrality and pathway enrichment provides a robust rationale for selecting these natural compounds as mechanistically active candidates for mitigating neuroinflammation.

Consistent with our bioinformatic projections, the experimental findings in BV2 murine microglial cells provide empirical evidence for the anti-inflammatory efficacy of Quercetin. In our model, Quercetin demonstrated a benign safety profile and significantly blunted the LPS-triggered transcriptional upregulation of IL-1β, TNF-α, IL-6, and iNOS. While simultaneously elevating the reparative mediator IL-10. Mechanistically, this pharmacological intervention was underpinned by the suppression of RAGE expression and the subsequent inhibition of P65 and IκB phosphorylation, confirming the disruption of the RAGE/NFκB axis [30,43,45]. The selection of a pretreatment regimen in this study was based on the pharmacokinetic profile of dietary flavonoids, which typically achieve steady-state concentrations in vivo prior to inflammatory challenges [40]. This prophylactic design allowed us to proactively assess whether Quercetin could intercept the RAGE-dependent inflammatory cascade at the transcriptional level, effectively preventing the recruitment of NF-κB to proinflammatory gene promoters [45]. By introducing the compound before the inflammatory trigger, we demonstrated that its inhibitory effect on RAGE and downstream NF-κB activation is a direct pharmacological consequence rather than a post-inflammatory neutralization [28]. However, while the LPS-stimulated BV2 microglial model is widely utilized for investigating neuroinflammation, we acknowledge that it does not directly represent chronic insomnia. Consequently, our current experiments primarily demonstrate Quercetin’s anti-inflammatory properties rather than direct anti-insomnia activity [28].

Beyond the in vitro constraints, this hypothesis-generating study shares several broader limitations [44]. First, the six hub genes and the proposed microbiota-substrate-metabolite-target network (as depicted in Figure 8) were identified entirely through computational database mining and network topological analysis [23]. Because we did not perform direct multi-omics evaluations—such as 16S rRNA microbiome sequencing, targeted metabolomics, or clinical cohort validation—the regulatory relationships proposed herein must be strictly interpreted as predictive associations rather than experimentally confirmed interactions. Second, the biological relevance of the computationally identified hub genes to CI relies largely on prediction and lacks direct physiological validation in our current study. Furthermore, this study did not evaluate specific sleep-related physiological metrics (such as sleep latency, total sleep duration, or EEG/EMG-based sleep architecture) [1,2]. Therefore, our data primarily establish an anti-inflammatory effect, and any conclusions regarding the direct improvement of insomnia symptoms must be interpreted with extreme caution. Future work must prioritize the experimental validation of these hub genes and predicted interactions using targeted multi-omics in disease-relevant patient cohorts and in vivo animal sleep models to explicitly assess target engagement and therapeutic impacts.

## 5. Conclusions

In summary, our integrated computational framework—anchored by network pharmacology and molecular docking, and further validated through in vitro assays—suggests that dietary phytochemicals (such as quercetin) and gut microbiota-driven biotransformation can modulate the inflammatory and immuno-metabolic pathways associated with chronic insomnia (CI). This modulation is primarily mediated via inflammatory and immuno-metabolic signaling axes, specifically encompassing the TNF/NF-κB and AGE/RAGE pathways. Notably, Quercetin and other phenolic derivatives emerged as prominent multi-target candidates with the potential to mitigate CI-associated neuroinflammation. Specifically, Quercetin demonstrated potent anti-neuroinflammatory effects in BV2 microglial models by successfully intercepting the RAGE/NF-κB signaling axis. However, because the in vitro model primarily represents neuroinflammation and no direct sleep parameters were evaluated, these findings demonstrate an anti-inflammatory action rather than direct anti-insomnia activity. Furthermore, given that the hub targets and molecular docking results are fundamentally predictive, these network associations require rigorous in vivo and clinical validation. Thus, our results should be viewed as providing a pathophysiological bridge for inflammation management rather than a direct confirmation of insomnia reversal. Collectively, this study establishes a robust mechanistic foundation for future experimental validation and comprehensive safety assessments of diet- and microbiome-targeted strategies for managing the systemic burden of CI.

## Figures and Tables

**Table 1:** Bioinformatic resources, software tools, and computational analysis platforms utilized in the study.

NO	Database, Software, and Analysis Platform	Website	Version
1	ADMETlab	https://admetlab3.scbdd.com (accessed on 25 July 2026).	v3.0
2	Wei Sheng Xin	https://bioinformatics.com.cn/ (accessed on 25 July 2026)	\
3	CTD	https://ctdbase.org/ (accessed on 25 July 2026)	Revision18079
4	Cytoscape software	https://cytoscape.org/ (accessed on 25 July 2026)	v3.10.3
5	DAVID Bioinformatics	https://davidbioinformatics.nih.gov/ (accessed on 25 July 2026)	v2025_2
6	Genecards	https://www.genecards.org/ (accessed on 25 July 2026)	v5.26
7	gutMGene	https://bio-computing.hrbmu.edu.cn/gutmgene/ (accessed on 25 July 2026)	v2.0
8	OMIM	https://www.omim.org/ (accessed on 25 July 2026)	\
9	PubChem	https://pubchem.ncbi.nlm.nih.gov/ (accessed on 25 July 2026)	\
10	R4.52.2	https://www.r-project.org/ (accessed on 25 July 2026)	\
11	RStudio	https://posit.co/products/open-source/rstudio (accessed on 25 July 2026)	v2026.01.1+403
12	Similarity ensemble approach	https://sea.bkslab.org/ (accessed on 25 July 2026)	\
13	STRING	https://string-db.org/ (accessed on 25 July 2026)	V12.0
14	Swiss Target Prediction	https://swisstargetprediction.ch/ (accessed on 25 July 2026)	\
15	SwissADME	https://swissadme.ch/ (accessed on 25 July 2026)	\

**Table 2:** Primer sequences for qRT-PCR analysis.

Gene	Forward Primer (5′-3′)	Reverse Primer (5′-3′)
*GAPDH*	CATGGCCTTCCGTGTTCCTA	CCTGCTTCACCACCTTCTTGA
*TNF-α*	TCTTCTCATTCCTGCTTGTGG	ATGAGAGGGAGGCCATTTG
*iNOS*	GGGCAGCCTGTGAGACCTT	TGAAGCGTTTCGGGATCTG
*IL-1β*	CCCAAGCAATACCCAAAGAA	GCTTGTGCTCTGCTTGTGAG
*IL-6*	CAAAGCCAGAGTCCTTCAGAG	AGCATTGGAAATTGGGGTAG
*IL-10*	CAAGGAGCATTTGAATTCCC	GGCCTTGTAGACACCTTGGTC

**Table 3:** Molecular docking scores and binding affinities between core metabolites and key targets.

Targets	Compound	CDOKER ENERGY	Targets	Compound	CDOKER ENERGY	Targets	Compound	CDOKER ENERGY
PPARG	Quercetin	−30.5785	IL1β	Quercetin	24.6605	AKT1	Quercetin	−37.0513
	3-(4-Hydroxyphenyl) propionic acid	−24.7837		3-(4-Hydroxyphenyl) propionic acid	−21.5831		3-(4-Hydroxyphenyl) propionic acid	−28.2678
	Daidzein	−22.8037		Daidzein	−17.8125		Daidzein	−26.1061
	Dihydrocaffeic acid	−28.1556		Dihydrocaffeic acid	−24.6923		Dihydrocaffeic acid	−32.2308

Note: CDOKERENERGY: kcal/mol, PPARG (8B95), IL-1β (6Y8I), and AKT1 (3CQW).

**Table 4:** Pharmacokinetic assessment and drug-likeness profiling of candidate metabolites.

Compound	MW	HBA	HBD	MLOGP	Lipinski’s Violations	Bioavailability Score	TPSA
Quercetin	302.24	7	5	−0.56	0	0.55	131.36
3-(4-Hydroxyphenyl) propionic acid	166.17	3	2	1.37	0	0.85	57.53
Daidzein	254.24	4	2	1.08	0	0.55	70.67
Dihydrocaffeic acid	182.17	4	3	0.79	0	0.56	77.76

**Note:** The following criteria were applied for drug-likeness evaluation: **MW** (molecular weight, ≤500 Da); **HBA** (number of hydrogen bond acceptors, ≤10); **HBD** (number of hydrogen bond donors, ≤5); **MLOGP** (Moriguchi octanol-water partition coefficient, ≤4.15). Adherence to Lipinski’s violations refers to the count of rules breached within the Rule of Five framework. Bioavailability Score was benchmarked at ≥0.55, and **TPSA** (topological polar surface area) was restricted to ≤140 A^2^.

**Table 5:** The evaluation of toxicity on key metabolites.

Compound	hERG Blockers	H-HT	DILI	Neurotoxicity-DI	Carcinogenicity
Quercetin	Non-blocker	negative	positive	negative	positive
3-(4-Hydroxyphenyl) propionic acid	Non-blocker	negative	negative	negative	negative
Daidzein	Non-blocker	negative	negative	negative	positive
Dihydrocaffeic acid	Non-blocker	negative	negative	positive	negative

**Note: hERG Blockers:** human Ether-à-go-go-Related Gene blockers (potential for cardiac QT prolongation). **H-HT:** Human Hematotoxicity. **DILI:** Drug-Induced Liver Injury. **Neurotoxicity-DI:** Drug-Induced Neurotoxicity. **Carcinogenicity:** Potential to cause cancer.

## Data Availability

The datasets generated and analyzed during the current study are available from the corresponding author upon reasonable request.

## Supplementary Materials

The following supporting information can be downloaded at: https://www.mdpi.com/article/10.3390/biomedicines14081766/s1, Table S1: The information of 5 clusters. Table S2: 55 targets’ information. Table S3: Supplementary file of docking simulation.

## Abbreviations

The following abbreviations are used in this manuscript:

AGE	Advanced Glycation End-products
AKT1	AKT Serine/Threonine Kinase 1
BE	binding energy
BP	Biological Process
CC	Cellular Component
CI	chronic insomnia
CTD	Comparative Toxicogenomics Database
EPC	Edge Percolated Component
GO	Gene Ontology
GMFA	GeneMANIA functional association
GM	Gut microbiota
IL-1β	Interleukin 1 Beta
IL-6	Interleukin 6
IL-17	Interleukin-17
KEGG	Kyoto Encyclopedia of Genes and Genomes
LPS	Lipopolysaccharide
MCC	Maximal Clique Centrality
MNC	Maximum Neighborhood Component
MF	Molecular Function
NF-κB	Nuclear Factor Kappa B
PPARG	Peroxisome Proliferator Activated Receptor Gamma
PI3K	Phosphoitide 3-Kinase
PPI	protein–protein interaction
RAGE	Receptor for Advanced Glycation End-products
SEA	Similarity Ensemble Approach
STP	SwissTargetPrediction
TLR	Toll-like receptor
TNF	Tumor Necrosis Factor
TP53	Tumor Protein P53
